# Geographic characteristics of HTLV-1 molecular subgroups and genetic substitutions in East Asia: Insights from complete genome sequencing of HTLV-1 strains isolated in Taiwan and Japan

**DOI:** 10.1371/journal.pntd.0011928

**Published:** 2024-02-05

**Authors:** Satoshi Nozuma, Akiko Yoshimura, Shun-Chung Pai, Hung-Jen Chen, Eiji Matsuura, Masakazu Tanaka, Daisuke Kodama, Mika Dozono, Toshio Matsuzaki, Hiroshi Takashima, Ya-Chien Yang, Ryuji Kubota

**Affiliations:** 1 Department of Neurology and Geriatrics, Kagoshima University Graduate School of Medical and Dental Sciences, Kagoshima, Kagoshima, Japan; 2 Division of Quality, Taipei Blood Center, Taipei, Taiwan; 3 Department of Clinical Laboratory Sciences and Medical Biotechnology, National Taiwan University College of Medicine, Taipei, Taiwan; 4 Division of Neuroimmunology, Joint Research Center for Human Retrovirus Infection, Kagoshima University, Kagoshima, Japan; Walter and Eliza Hall Institute of Medical Research, AUSTRALIA

## Abstract

**Background:**

Although Japan is a major endemic area for human T-lymphotropic virus type 1 (HTLV-1) and the virus has been well-studied in this region, there is limited research on HTLV-1 in surrounding regions. In this study, we determined the complete genome sequences of HTLV-1 strains isolated from Taiwan and Japan and investigated the geographic characteristics of molecular subgroups and substitution mutations to understand the spread of HTLV-1 and its correlation with human migration.

**Methodology/Principal findings:**

The complete genome sequences of 26 HTLV-1 isolates from Taiwan were determined using next-generation sequencing and were compared with those of 211 isolates from Japan in terms of subgroup and genetic mutations. In total, 15/26 (58%) isolates from Taiwan belonged to the transcontinental subgroup and 11/26 (42%) isolates belonged to the Japanese subgroup. The transcontinental subgroup was significantly more prevalent among Taiwanese isolates than Japanese isolates (58% vs 18%, *P* < 0.0001). The mutation rate for the complete HTLV-1 sequence was as low as 0.2%. On examining individual base substitutions, the G-to-A mutation was predominant. Bayesian phylogenetic tree analysis estimated the time to the most recent common ancestor for the transcontinental and Japanese subgroups to be 28447 years. The transcontinental subgroups from Taiwan and Japan appeared to form clusters according to their respective regions.

**Conclusions/Significance:**

The transcontinental subgroup of HTLV-1 is predominant in Taiwan, while the Japanese subgroup is common in Japan. The difference in subgroup distribution may be attributed to the initial spread of the transcontinental subgroup in East Asia, followed by the influx of the Japanese subgroup.

## Author summary

Human T-lymphotropic virus type 1 (HTLV-1) is a retrovirus that infects 5–10 million people worldwide. HTLV-1 infection can cause serious diseases including hematologic malignancy, adult T-cell leukemia/lymphoma (ATL), and progressive neurologic disease known as HTLV-1-associated myelopathy/tropical spastic paraplegia (HAM/TSP). An increase in the mortality rate among HTLV-1-infected individuals has become an issue of concern in recent years. Differences in HTLV-1 infection rates and subgroup distribution are considered to be caused by the historical global movement of HTLV-1-infected individuals. Furthermore, recent global population movements have resulted in the redistribution of HTLV-1-infected individuals, which may lead to outbreaks of ATL and HAM/TSP in non-endemic areas. In this study, we investigated the molecular characteristics of HTLV-1 in East Asia and found that the transcontinental subgroup was more prevalent in Taiwan, whereas the Japanese subgroup was common in Japan. The difference in subgroup distribution may be attributed to the initial spread of the transcontinental subgroup in East Asia, followed by the influx of the Japanese subgroup.

## Introduction

Human T-lymphotropic virus type 1 (HTLV-1) was the first human retrovirus to be discovered and infects 5–10 million people worldwide [1]. The majority of infected individuals remain asymptomatic carriers (ACs) for the rest of their lives, whereas 2%–5% develop adult T cell leukemia/lymphoma (ATL) [2] and another 0.25%–3.8% develop HTLV-1-associated myelopathy/tropical spastic paraparesis (HAM/TSP) [3,4]. HTLV-1 infection occurs worldwide with uneven infection rates [5]. Highly endemic areas are the Caribbean, Central and South America, Central and West Africa, the Middle East, Australia and Melanesia, and southwest Japan. HTLV-1 transmission occurs mainly via three routes: vertical transmission, sexual contact, and contaminated blood products.

HTLV-1 is a genetically stable retrovirus and is classified into seven primary genetic subtypes (a-g) based on the nucleotide diversity observed in its long terminal repeat (LTR) region [6]. Among these, the cosmopolitan subtype can be further categorized into five subgroups: (A) transcontinental, (B) Japanese, (C) West African, (D) North African, and (E) Peruvian Black [7,8]. The transcontinental subgroup is most widespread worldwide and the other subgroups have distinct geographic distributions. The distribution pattern is considered to reflect the global movements of ancient infected populations. In East Asia, the coexistence of transcontinental and Japanese subgroups has been observed based on genetic analysis of LTR and envelop sequences of the HTLV-1 genome [9–11]. The transcontinental subgroup is predominant in China and Taiwan, while the Japanese subgroup is prevalent in Japan.

Recent rapid globalization has caused a redistribution of HTLV-1 infection due to immigration and migration from endemic regions to urban areas [12]. It is now possible to encounter a case with ATL or HAM/TSP even in non-endemic areas. Meta-analysis revealed that HTLV-1 infection itself increased mortality [13]. HTLV-1 infection still significantly impacts global health and the World Health Organization has warned against HTLV-1 [14].

In this study, we sequenced the complete genomes of HTLV-1 strains isolated from Taiwan and Japan and investigated the geographic characteristics of molecular subgroups and substitution mutations to understand the spread of HTLV-1 and its correlation with human migration. The transcontinental subgroup was significantly more prevalent in Taiwan than in Japan. Furthermore, the mutation rate for the complete HTLV-1 genome sequence was as low as 0.2%, with the G-to-A mutation being the predominant type of base substitutions. Bayesian phylogenetic tree analysis showed that the transcontinental subgroup formed distinct clusters for each respective region, whereas the Japanese subgroup included a mix of sequences from both countries. The difference in subgroup distribution may be due to the initial spread of the transcontinental subgroup in East Asia, followed by the influx of the Japanese subgroup.

## Methods

### Ethics statement

The study was approved by the Institutional Review Board of Kagoshima University (No. 170207EKI) and National Taiwan University (No. 201707030RINA). All participants provided written informed consent prior to participation in accordance with the Declaration of Helsinki.

### Study population

A total of 26 samples from Taiwan and 211 samples from Japan were included in this study. HTLV-1 sequence data from Japanese samples were obtained from our previous study [15].

### Preparation and sequencing of the complete provirus genomes

DNAs from 26 samples from Taiwan, obtained from the Taipei Blood Center and National Taiwan University, were used in the present study. The complete provirus genome was amplified using nested PCR as previously reported [15,16]. Initially, we amplified two overlapping fragments of the HTLV-1 gene from genomic DNA. Subsequently, PCR products were subjected to nested PCR. The final PCR products were purified with the MinElute PCR purification kit (Qiagen, Hilden, Germany). All PCR libraries were ligated with specified indexes and simultaneously sequenced on the MiSeq system in accordance with the manufacturer’s protocol (Illumina, California, USA). Due to the limited volume of samples preserved in Taiwan, we could not measure the HTLV-1 proviral load in samples from Taiwan in this study.

### Complete sequence determination and phylogenetic tree analysis

The raw sequencing reads were processed using CLC genomics Workbench v 22.0 (CLC bio, Aarhus, Denmark). The reads were trimmed using the quality score limit of 0.05 and a maximum of two ambiguous nucleotides. Subsequently, the reads were mapped to the HTLV-1 complete genome (AB513134), allowing up to 2 mismatches. All bases of the alignment were examined and single nucleotide polymorphisms were detected using the Fixed Ploidy Variant Detection tools. All comparison alignments were conducted and a phylogenetic tree was constructed by the neighbor-joining method with 1000 bootstrap replications using Jukes-Cantor model. The neighbor-joining method was used due to its efficiency with large datasets and its capability to construct trees without the assumption of constant evolutionary rates. Seven complete HTLV-1 sequences, including two transcontinental subgroups (RK13 and BOI), three Japanese subgroups (ATK, ATL-YS, and AB513134), one Central African subtype (SF26), and one Melanesian subtype (MEL5), were used to construct the phylogenetic tree. To identify possible mutations in the transcontinental subgroup, sequences belonging to the transcontinental subgroup were realigned to a common reference sequence created from 37 samples of transcontinental subgroups as previously reported [15]. HTLV-1 reference sequences were retrieved from the GenBank database.

### Molecular evolution analysis

Time-scaled tree and evolutionary analyses were conducted using the Markov chain Monte Carlo (MCMC) method implemented in Bayesian Evolutionary Analysis Sampling Trees 2 (BEAST 2, v 2.7.4) [17]. The evolution and transmission of HTLV-1 in Taiwan and Japan were analyzed using *env* sequences obtained in this study and existing reference sequences from Brazil in the GenBank database. The following parameters were used for the analysis: the GTR model of nucleotide substitution, the Strict clock model, the Yule process model, and a substitution rate of 2.1× 10^˗7^ (2.1× 10^˗8^ to 4.5× 10^˗7^), as previously described [9,18]. The lengths of the MCMC were set to 100 million generations and trees were collected every 10,000 steps. Convergence of the MCMC was calculated using the program Tracer v1.7.2 and confirmed the effective sampling size to be ≥ 200. TreeAnnotator was used to analyze the maximum clade credibility tree with common ancestor heights following the burn-in of the first 10% sampled trees.

### Statistical analysis

An unpaired *t*-test was used to compare the number of mutations and specifically G-to-A mutations between HTLV-1 sequences from Taiwan and Japan. The frequency of mutations in the HTLV-1 genome was analyzed by one-way repeated measures analysis of variance followed by Dunnett’s multiple comparison test. A chi-square test was used to compare the frequency of the transcontinental subgroup between Taiwan and Japan. All statistical analyses were performed using Prism version 9.5.1 (GraphPad software). *P* < 0.05 was considered statistically significant. Values are presented as the mean and 95% confidence intervals (CI).

## Results

### Phylogenetic analysis of HTLV-1 strains from Taiwan and Japan

The complete genome sequences of 26 HTLV-1 isolates from Taiwan and 211 HTLV-1 isolates from Japan were analyzed in this study. The clinical characteristics of the subjects from which they were isolated are listed in Table 1. The subjects from Taiwan included 21 ACs, 2 HAM/TSP patients, and 3 patients with other hematologic diseases, while the subjects from Japan included 89 ACs and 122 HAM/TSP patients.

**Table 1:** Characteristics of the subjects.

Disease	Taiwan (N = 26)	Japan (N = 211)
Asymptomatic carriers	21	89
HAM/TSP	2	122
Aplastic anemia	1	0
Acute myeloid leukemia	1	0
Hodgkin lymphoma	1	0

HAM/TSP: HTLV-1-associated myelopathy/tropical spastic paraparesis.

**Fig 1:**
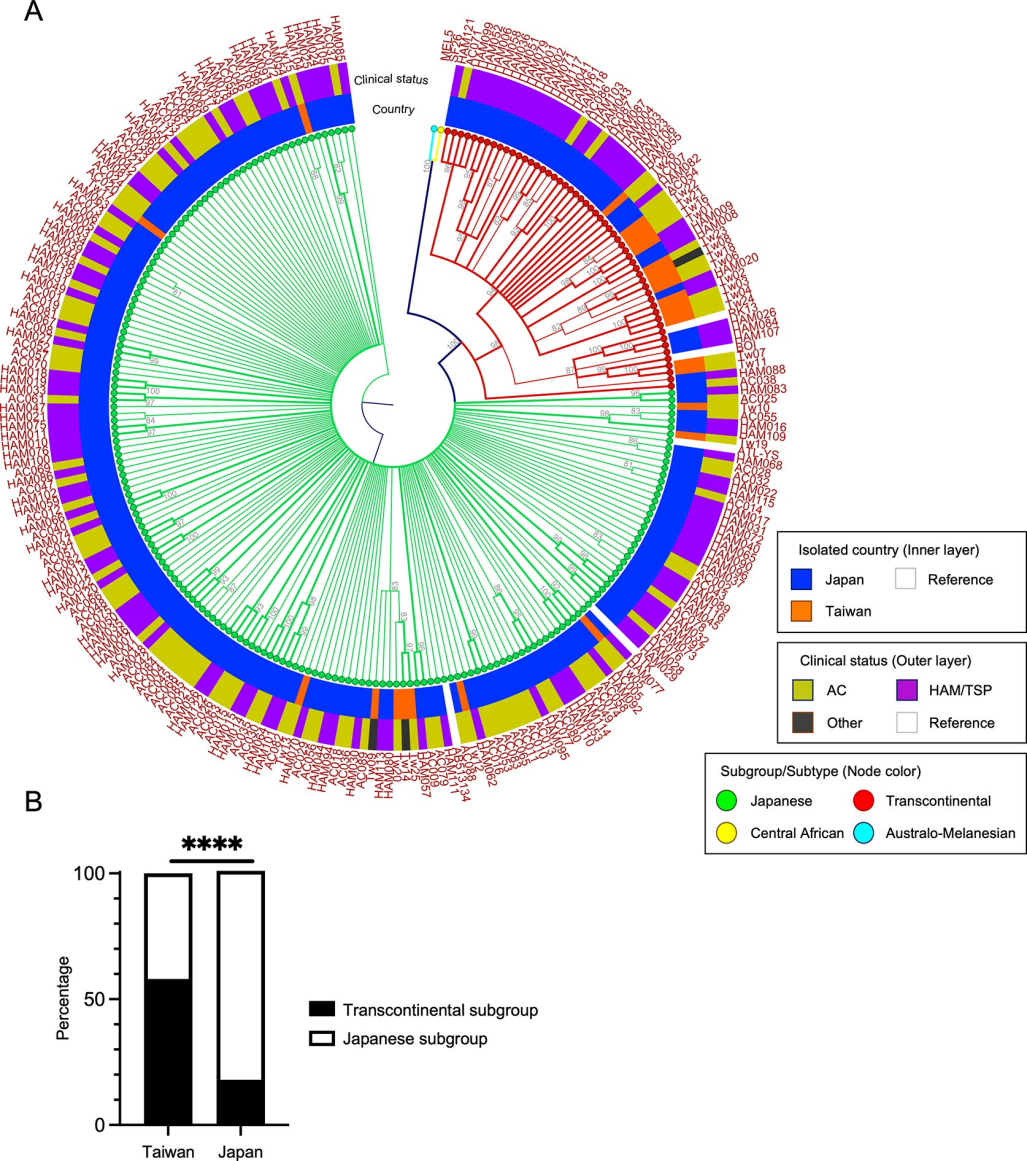
Phylogenetic analysis of HTLV-1 based on the complete genome sequences of isolates from Taiwan and Japan. **(A)** Phylogenetic tree generated by the neighbor-joining method with 1000 bootstrap replications using isolated 26 HTLV-1 sequences isolated from Taiwan and 211 from Japan. Nodes represent the HTLV-1 subgroup. The inner layer represents the country of isolation or the reference. The outer layer represents clinical status. The names of isolates are shown on the outside of the layer. Only bootstrap values >70 are shown. **(B)** Comparison of the frequency of the transcontinental subgroup of HTLV-1 between isolates from Taiwan and Japan. ****P < 0.0001.

To determine the individual HTLV-1 genome sequences, reads obtained from MiSeq were mapped and assembled to the reference sequence of AB513134, as previously reported [15]. On average, 466,375 reads were obtained with a coverage of 4,370, and the alignments of all samples achieved a minimum coverage of 20 bp. A consensus sequence for each sample was extracted and analyzed for subgroup and genetic mutations. The mean length of consensus sequences was 9033 (95% CI 9033 to 9034). We conducted phylogenetic analysis using these whole genome sequences (Fig 1A and the names of isolates and accession numbers on GenBank are listed in S1 Table). All of the samples from Taiwan and Japan belonged to the Cosmopolitan subtype of HTLV-1. Subgroup analysis revealed that 15/26 (58%) HTLV-1 isolates from Taiwan belonged to the transcontinental subgroup and 11/26 (42%) isolates belonged to the Japanese subgroup (Fig 1B). The transcontinental subgroup was significantly more common among the Taiwanese isolates than the Japanese isolates (58% vs 18%; *P* < 0.0001, Fig 1B). In addition, the HTLV-1 isolates from two Taiwanese patients with HAM/TSP were classified as belonging to the transcontinental subgroup. Examining the distribution based on clinical status, no predominant clusters were observed (outer layer in Fig 1A).

### HTLV-1 sequence mutation analysis

**Fig 2:**
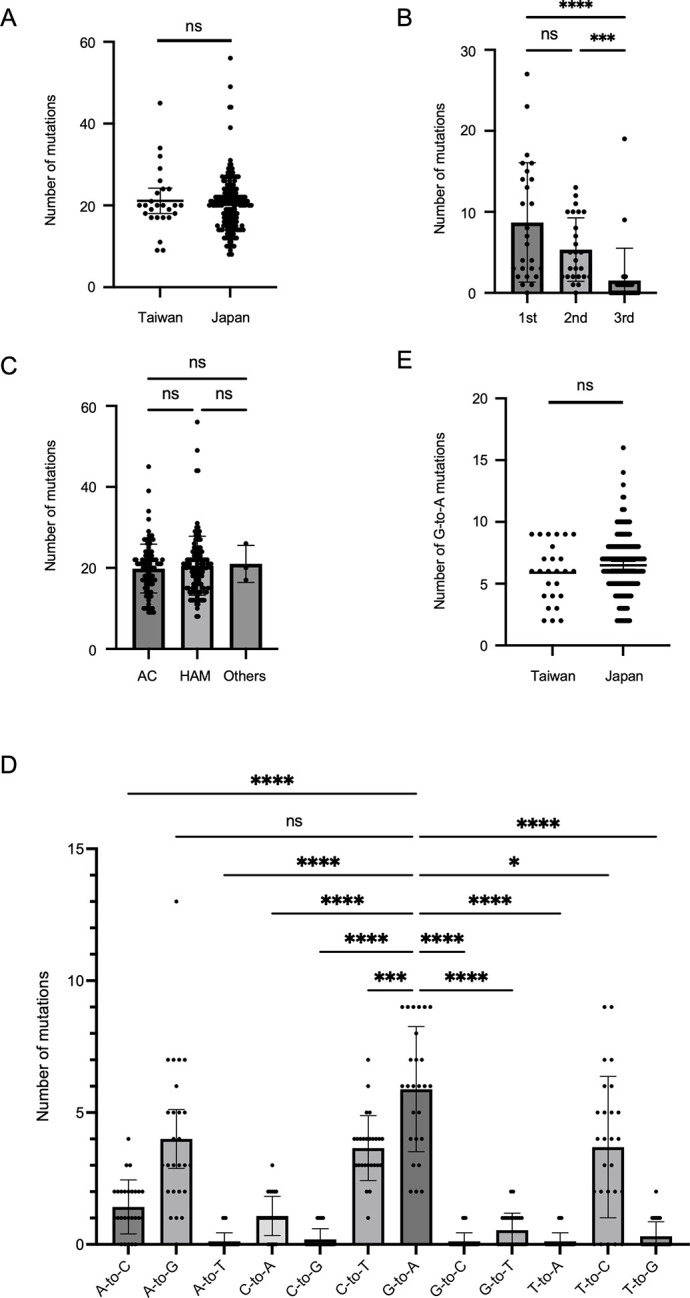
Frequency of mutation in the complete HTLV-1 genome sequences. **(A)** Comparison of the number of mutations in the genome sequences between HTLV-1 isolates from Taiwan and Japan. The horizontal bars indicate the mean and 95% confidence intervals (CI). Comparison of the number of mutations among codon positions **(B)** and clinical status **(C)**. The columns and vertical bars indicate the mean and 95% CI. **(D)** Comparison of the number of mutations per base substitution in the samples from Taiwan. **(E)** Comparison of the number of G-to-A mutations between HTLV-1 isolates from Taiwan and Japan.

HTLV-1 has low genetic variability. We examined whether there were differences in genetic variation between isolates from Taiwan and Japan using whole HTLV-1 genome sequencing. The distribution of nucleotide bases in the HTLV-1 sequence (AB513134) is as follows: A (23.1%), C (35.0%), G (18.9%), and T (23.0%). To correct for differences by subgroup, the transcontinental and Japanese subgroups were mapped to the transcontinental and Japanese reference (AB513134) sequences, respectively. The mean number of mutations was 21.1 (0.23%, 95% CI 18.0 to 24.2) for isolates from Taiwan and 20.1 (0.22%, 95% CI 19.2 to 21.0) for isolates from Japan, with no difference between the two groups (Fig 2A). When comparing mutations in the coding region, the number of mutations was significantly higher in the 1st and 2nd codon positions than in the 3rd position (Fig 2B). In contrast, for the HBZ antisense strand, mutation frequency did not vary according to the position within the codon (S1 Fig). The type of mutations in the coding regions is listed in S2 Table. Moreover, the number of mutations did not differ among clinical statuses, including AC and HAM/TSP (Fig 2C).

Because base substitutions in HTLV-1 sequences have been reported to have deviated [15,16], we examined variations in the present samples and found that the G-to-A mutation was the predominant base substitution, except for the A-to-G mutation (Fig 2D). The number of G-to-A mutations was examined in the Taiwanese and Japanese samples; however, there was no difference between the two groups (Fig 2E). Amino acid mutations in the coding regions of the HTLV-1 genome are listed in S3 Table. Abortive genetic changes were detected exclusively in the transcontinental subtype.

### Evolution analysis

**Fig 3:**
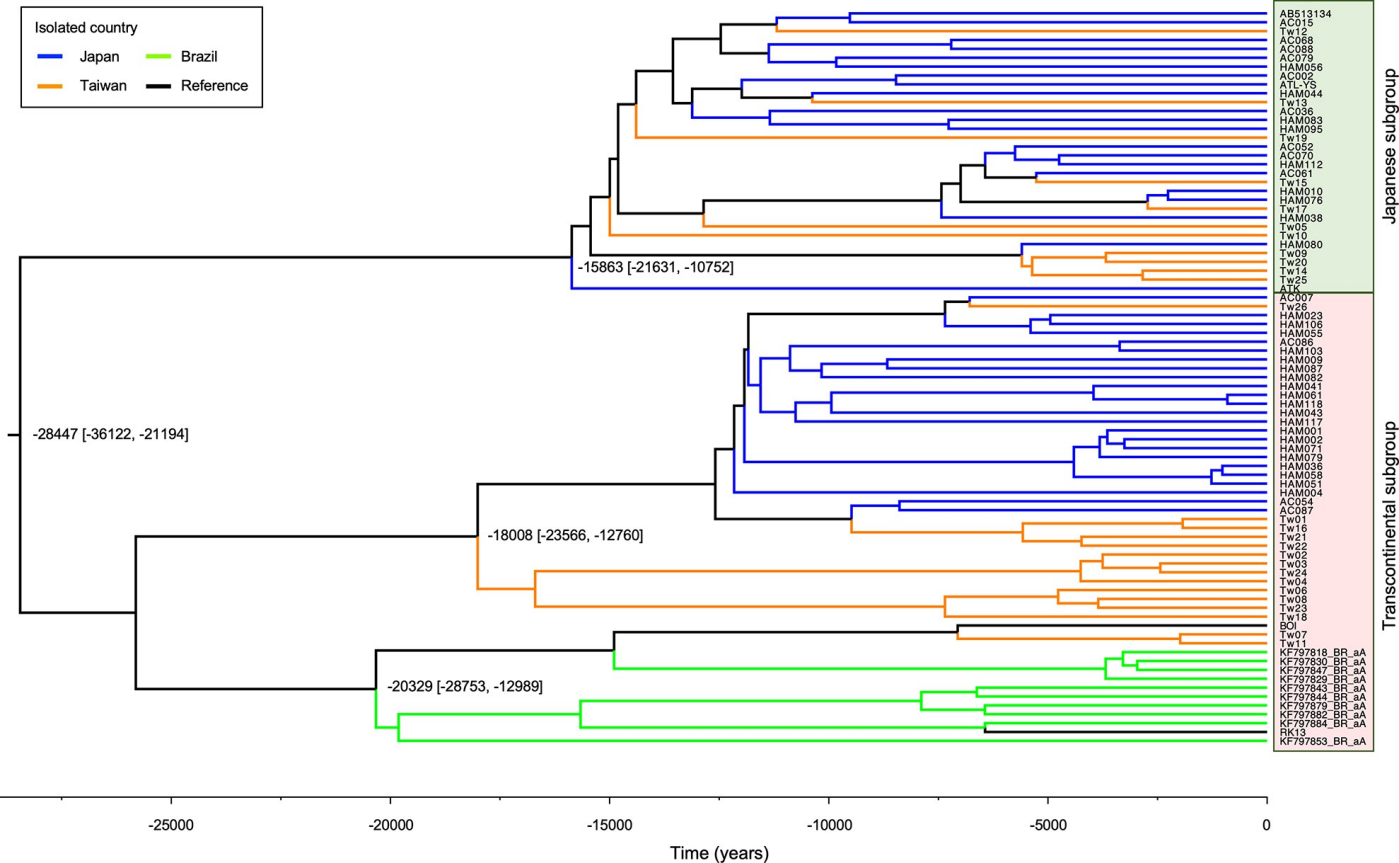
Bayesian phylogenetic tree based on envelope (*env*) sequences. Branches are colored according to the country of isolation. The time scale runs from the most recent common ancestor to 2023. The numbers on each node refer to the mean years (95% highest posterior density interval) and only posterior values of >0.9 are shown.

To examine the origin of HTLV-1 in Taiwan and Japan, Bayesian phylogenetic tree analysis of *env* sequences was performed using models previously reported for the evolution of HTLV-1 sequences [9,18]. HTLV-1 strains were divided into two major subclades, the transcontinental and the Japanese subgroups, according to the phylogenetic tree (Fig 3). The time to the most recent common ancestor (tMRCA) of these two major subclades was estimated to be 28,447 years ago. Isolates of the transcontinental subgroup from Taiwan, Japan, and Brazil, appear to form clusters according to their respective region of origin, whereas isolates of the Japanese subgroup from Taiwan and Japan appear to be distributed across the tree.

## Discussion

Genome sequence analysis of HTLV-1 isolates in this study revealed that the transcontinental subgroup was prevalent in Taiwan, whereas the Japanese subgroup was common in Japan. Within East Asia, HTLV-1 is endemic in Japan, but its prevalence in neighboring regions, such as Korea and China, is low. In Taiwan, a moderate infection rate of 0.06% has been reported based on screening tests of blood donors [19]. The distribution of HTLV-1 subgroups also differs, with the Japanese subgroup being predominant in Japan, whereas the transcontinental subgroup is common in China and Taiwan [9,10]. In our study, 58% of isolates from Taiwan were classified as belonging to the transcontinental subgroup, which was significantly higher than the 18% detected for isolates from Japan (Fig 1B). The frequency of the transcontinental subgroup in China is approximately 71% [9], and the prevalence of the transcontinental subgroup decreases eastward moving from China through to Taiwan and Japan.

Differences in the distribution of HTLV-1 subgroups are considered to be caused by the ancient global movement of HTLV-1-infected individuals. More than two Paleo-Mongoloid lineages possibly migrated to East Asia in the Paleolithic period, resulting in a present coexistence of the two HTLV-1 subgroups [20]. In Japan, the transcontinental subgroup is predominantly found in regions such as Ryukyu and Ainu, which are located at the periphery of the country, while the Japanese subgroup is distributed closer to the mainland, known as Honshu [21,22]. This suggests that the transcontinental subgroup may have initially entered the country, followed by the Japanese subgroup [22]. The phylogenetic tree constructed by the Bayesian method revealed that the transcontinental subgroup formed distinct clusters corresponding to Taiwan and Japan, while the Japanese subgroup included a mixture of sequences from both countries (Fig 3). Since the low sequence variation is likely to indicate a genotype derived from a common ancestor, the results suggest that the transcontinental subgroup entered Taiwan and Japan earlier and formed distinct clusters in each region, whereas the Japanese subgroup entered later and dispersed. Further studies on the distribution of HTLV-1 subgroups in the broader East Asian region may provide valuable insights into the spread of HTLV-1 and human migration patterns during ancient history.

Most studies have been unable to identify an association between mutations in HTLV-1 and HTLV-1-related diseases [15,23,24]. However, the transcontinental subgroup of HTLV-1 has been reported to be detected more frequently in HAM/TSP patients compared with ACs [11,15]. In this study, viral isolates from the two cases of HAM/TSP in Taiwan belonged to the transcontinental subgroup. PVL is generally higher in patients with HAM/TSP, however, studies have indicated no difference in PVL between the transcontinental and Japanese subgroups [15,25]. This suggests that factors other than the HTLV-1 subgroup contribute to elevated PVL in HAM/TSP. Patients with HAM/TSP that harbor the transcontinental subgroup of HTLV-1 are reported to exhibit lower levels of HBZ mRNA expression [25] and higher levels of CXCL10, which has been suggested as a potential prognostic biomarker for HAM/TSP [26]. Different subgroups of HTLV-1 have been found to exhibit distinct patterns of host gene expression [26], which could potentially contribute to the increased incidence of HAM/TSP in individuals infected with the transcontinental subgroup. This study identified only two patients with HAM/TSP, therefore further studies are needed to verify whether the transcontinental subgroup is more frequent in patients with HAM/TSP in Taiwan.

The HTLV-1 genome is thought to be highly conserved and this was confirmed by the results of our whole genome sequencing study that revealed a mutation rate of as low as 0.2%. On examining individual base mutations, the G-to-A mutation was found to be significantly more common than other types of base mutations with the exception of A-to-G mutation. The high occurrence of the G-to-A mutation has been attributed to the host defense gene APOBEC3G, which induces the deamination of cytosine residues during reverse transcription in retroviruses [27,28]. Previous studies have demonstrated that G-to-A mutations are abundant in HTLV-1 proviruses, and in addition, abortive genetic changes including deletions, insertions, and nonsense mutations have been frequently identified in patients with ATL [16], but less frequently identified in patients with HAM/TSP and ACs [15]. The limited detection of abortive genetic changes may be attributed to the absence of patients with ATL in our study. The A-to-G mutation was also frequently observed in this study. A previous report has similarly shown that the A-to-G mutation is particularly common in the transcontinental subgroup [15]. The reason for the increased prevalence of the A-to-G mutation remains unclear, but factors other than APOBEC3G might play a role. Furthermore, we used the same nucleotide substitution rate in *env* sequences as previously employed in the Bayesian evolutionary analysis of the HTVL-1 virus [9,18]. However, given a bias in base substitutions including an accumulation of the G-to-A, mutations might occur faster than anticipated. Although our analysis is not exhaustive, we have established the approximate estimated year of the most recent common ancestor of the transcontinental and the Japanese HTLV-1 subgroups.

Recent global migration has led to the redistribution of HTLV-1-infected individuals. This could be attributed to the movement of infected people from endemic areas to urban regions [12], as well as horizontal transmission among adolescents and young adults [29]. HTLV-1 can directly impact health by causing ATL, a hematologic malignancy, and HAM/TSP, a neurological intractable disease. A recent meta-analysis reported an association between HTLV-1 infection and an increase in all-cause mortality, as well as various other diseases [13]. A comprehensive investigation of infection rates and the prevalence of HTLV-1 subtypes in both endemic and non-endemic regions is crucial for the effective prevention of HTLV-1 infection on a global scale.

In conclusion, a comparison of subgroups using the complete genomic sequences of HTLV-1 revealed that the transcontinental subgroup is predominant in Taiwan, while the Japanese subgroup is common in Japan. These results provide essential information for monitoring future trends of HTLV-1 infection. The difference in subgroup distribution may be attributed to the initial spread of the transcontinental subgroup in East Asia, followed by the influx of the Japanese subgroup. Comprehensive research into the distribution of HTLV-1 subgroups across the broader East Asian region, along with a molecular evolution analysis using an unbiased model for HTLV-1 sequences, would be helpful in understanding the historical spread of HTLV-1 and human migration.

## Supporting information

S1 Fig Comparison of the number of mutations among codon positions in HBZ region. (TIFF)

S1 Table Isolate names and accession numbers on GenBank. (XLSX)

S2 Table Type of mutations in the coding regions among HTLV-1 isolates from Taiwan. (XLSX)

S3 Table Gene mutations among HTLV-1 isolates from Taiwan. (XLSX)
