# Supplementary figures and images for: Geographic characteristics of HTLV-1 molecular subgroups and genetic substitutions in East Asia: Insights from complete genome sequencing of HTLV-1 strains isolated in Taiwan and Japan
Source: PLoS Negl Trop Dis. 2024 Feb 5;18(2):e0011928. doi: 10.1371/journal.pntd.0011928 (PMC10868808; doi:10.1371/journal.pntd.0011928)

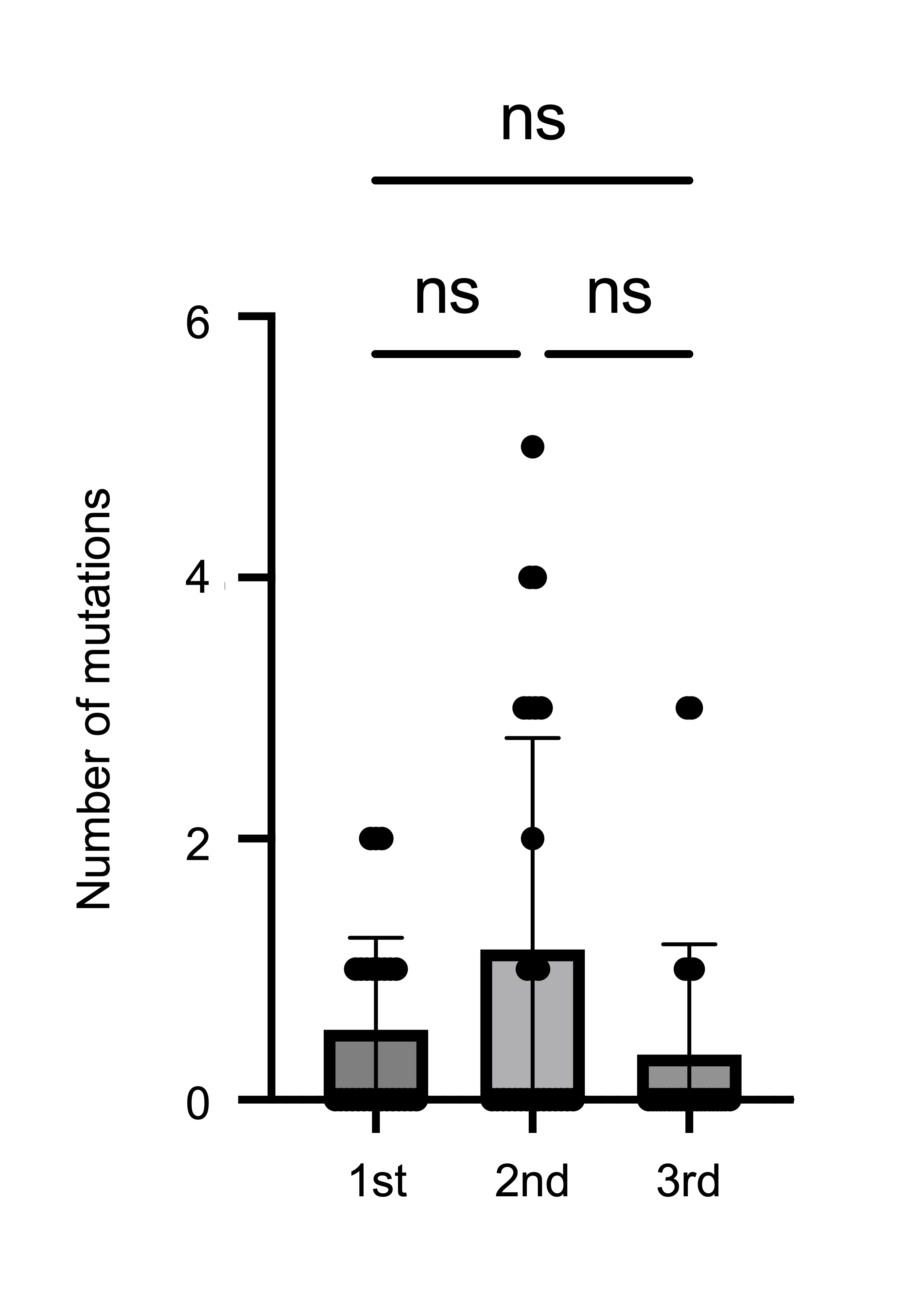

Supplement: S1 Fig — (TIFF) [file pntd.0011928.s001.tiff]
